# A scoping review of the use of visual tools and adapted easy-read approaches in Quality-of-Life instruments for adults

**DOI:** 10.1007/s11136-023-03450-w

**Published:** 2023-06-21

**Authors:** Rachel Milte, Digisie Jemere, Kiri Lay, Claire Hutchinson, Jolene Thomas, Joanne Murray, Julie Ratcliffe

**Affiliations:** Caring Futures Institute, College of Nursing and Health Sciences, Flinders University, GPO Box 2100, Adelaide, SA 5001 Australia

**Keywords:** Quality of life tools, Visual, Easy read, Adults, Patient reported outcome measures (PROMs)

## Abstract

**Purpose:**

Self-Reporting using traditional text-based Quality-of-Life (QoL) instruments can be difficult for people living with sensory impairments, communication challenges or changes to their cognitive capacity. Adapted communication techniques, such as Easy-Read techniques, or use of pictures could remove barriers to participation for a wide range of people. This review aimed to identify published studies reporting adapted communication approaches for measuring QoL, the methodology used in their development and validation among adult populations.

**Methods:**

A scoping review of the literature using the Preferred Reporting Items for Systematic Reviews and Meta-Analyses (PRISMA) extension for scoping reviews checklist was undertaken.

**Results:**

The initial search strategy identified 13,275 articles for screening, with 264 articles identified for full text review. Of these 243 articles were excluded resulting in 21 studies for inclusion. The majority focused on the development of an instrument (12 studies) or a combination of development with some aspect of validation or psychometric testing (7 studies). Nineteen different instruments were identified by the review, thirteen were developed from previously developed generic or condition-specific quality of life instruments, predominantly aphasia (7 studies) and disability (4 studies). Most modified instruments included adaptations to both the original questions, as well as the response categories.

**Conclusions:**

Studies identified in this scoping review demonstrate that several methods have been successfully applied e.g. with people living with aphasia post-stroke and people living with a disability, which potentially could be adapted for application with more diverse populations. A cohesive and interdisciplinary approach to the development and validation of communication accessible versions of QOL instruments, is needed to support widespread application, thereby reducing reliance on proxy assessors and promoting self-assessment of QOL across multiple consumer groups and sectors.

**Supplementary Information:**

The online version contains supplementary material available at 10.1007/s11136-023-03450-w.

## Background

For decades, there has been broad agreement among researchers, health professionals, policy makers and administrators regarding the importance of quality assessment across health and social care systems [1–3]. Despite this, there is still no single agreed ‘gold standard’ approach for measuring quality of care. Of the quality of care assessment models and frameworks that are available, the majority include some component of measuring the outcomes of care provided from the perspectives of care recipients themselves [1, 4]. In practice, clinical outcomes (e.g. physiologic markers, mortality or measures of morbidity) have taken the lead as the predominant approach for measuring care outcomes. Such indicators have come under criticism for potential difficulty in their interpretation and lack of meaning to the recipients of care themselves [1]. Additionally, clinical outcomes do not necessarily maintain their meaning and significance across multiple clinical populations. For example, weight loss among people experiencing overweight or obesity-related health conditions may be viewed positively. By comparison, weight loss among frail elderly people would be considered a poor outcome [5]. These challenges, along with the momentum gained from the social movement for involving patients and consumers in planning, managing and evaluating the health and social care they receive, has led to the rise of patient-reported outcome measures (PROMs) focused on quality of life (QOL) [6].

QOL is one of the most highly utilised PROMs in health research [7]. The maximisation of QOL for patients is acknowledged as the ultimate aim of health and social care [8]. By definition the measurement of QOL is subjective, incorporating the person’s own judgement of their current health and wellbeing in comparison with their expectations for those domains of their lives [9]. Increasingly there have been calls to include QOL as a key quality indicator across both health and social care settings [10]. Presently, a wealth of validated QOL instruments are available for application across a range of different health conditions and settings including generic QOL instruments (such as the EQ-5D-5L or WHOQOL suite of instruments) [7] and condition-specific instruments (for example the European Organisation for Research and Treatment of Cancer Quality of Life Questionnaire (EORTC QLQ-C30) or the Quality of Life in Alzheimer’s Disease (QOL-AD)) [7].

While instruments differ in composition, length and complexity, generally they take the form of text-based multiple-choice descriptive systems, requiring reading comprehension and written expression skills to complete. The vast majority of the commonly used instruments are not inclusive of people with diverse communication needs, low literacy or perceptual or cognitive difficulties [11]. Consequently, people with diverse communication needs are often excluded from QOL and outcomes research despite this population comprising a relatively large proportion of those utilising health and social care services [12–14]. The World Health Organization estimates over 1 billion people, equivalent to over 15% of the world’s population, live with a physical, sensory, intellectual or mental health impairment which impacts their daily lives [15]. Additionally, on average 20% of the population in OECD countries is either illiterate or has very low literacy skills, impacting on their ability to engage with institutional processes and health information in a written form [16]. The majority of older people receiving aged care services in Australia have some form of cognitive impairment or dementia [17, 18]. Thus, individuals who may find it difficult or impossible to complete traditional text-based PROMs are relatively common among those seeking and using health and social care services. In these settings, proxy assessment of QOL by family members or care providers is often used as the default option [19]. However, it is now acknowledged that proxy respondents are not a direct replacement for self-report of PROMs, due to evidence of systematic differences in the way that proxy assessors respond to questions about the QOL of the person compared to the person themselves [20, 21]. Empirical comparison studies incorporating self and proxy assessment generally find poor to moderate levels of agreement, especially for less easily observable psychosocial focused QOL domains as opposed to physical QOL domains. Generally, for populations of older people it has been found that overall QOL scores (either raw scores or scores converted into utilities) reported by proxy are lower than those reported by older people themselves, and that the difference in scores tends to increase over time [20]. These factors lead to a significant gap in our understanding and consequently our ability to provide high quality care services meeting the needs of the diverse population accessing health and social care services [12, 14, 22]. Presently the voices of large proportions of those accessing services (and particularly vulnerable to having poor QOL e.g. older adults with cognitive impairment and dementia, adults with intellectual impairments and/or sensory difficulties) are not being heard as we do not have appropriate communication accessible QOL instruments to facilitate self-reporting of QOL for quality assessment and evaluation in these populations [10].

Accessible communication techniques include, but are not limited to, pictures, pictograms, easy read or easy-English approaches, or modified layout or presentation of information. It is often assumed that people with communication difficulties, disability, or dementia are unable to speak for themselves [14, 23–25]. It is increasingly being recognised that such a diagnosis should not exclude a person from having the opportunity to fully contribute to evaluating the care they receive [26–28]. The onus is on the research community to develop better methods to facilitate the maximal inclusion of people who experience a range of cognitive and communication difficulties in self-assessment of their own QOL wherever possible. It has been identified that people with mild or moderate dementia can provide reliable answers on QOL instruments providing that the methods used to assess QOL support their communication needs [29]. Research has shown that adapted communication approaches, such as easy-read techniques, or visual representations of concepts can be successfully applied with groups including people living with intellectual disability, post-stroke aphasia, or culturally and linguistically diverse (CALD) background to support or replace written communication methods [30–32]. However, the extent to which these approaches have been successfully applied in existing QOL instruments is currently unknown.

Therefore, the aim of this review was to identify and describe existing QOL instruments which have used an adapted communication approach for use with adults. A secondary aim was to report the methods used in the development and validation of these instruments.

## Methods

A scoping review methodology was undertaken according to PRISMA extension guidelines [33, 34]. The protocol was prospectively registered with the Open Science Framework (https://doi.org/10.17605/OSF.IO/27RGS).

### Search strategy

A search strategy was developed in consultation with an academic librarian for databases including Medline, PubMed, Scopus, CINAHL, Emcare, Informit, PsycINFO, REHABDATA, Web of Science, Health and Social Science Instruments and Google Scholar. Searches were conducted including results up until November 2022 and no date limit was applied. Both subject heading and keyword searches were used where possible. Example search strategies can be found in Appendix 1.

### Eligibility criteria

Studies describing the development or validation of a QOL instrument including a significant component of accessible communication techniques with a focus on visual and/or an easy-read techniques to present the key information were included. Accessible communication techniques include, but are not limited to, pictures, pictograms, easy-read or easy-English approaches, or modified layout or presentation of information. We defined a significant component of accessible communication techniques as including modifications to two or more aspects of the instrument e.g. modification to the items of the instrument, or the response categories. Both generic and disease-specific instruments were eligible for inclusion. Studies reported in a language other than English and conference abstracts were excluded. Review articles were excluded but were hand searched for relevant articles. Studies where the target audience of the instrument, or a significant proportion of the sample (i.e. over 50%), were children or adolescents aged less than 18 years were also excluded. Children or adolescents may also benefit from adapted accessible communication versions of PROMs. However,their communication needs, brain development and cognitive processing are functionally and structurally different to those of adults, and potentially therefore we their needs could be quite different to that of an adult population. Therefore, we chose to focus on adult populations (aged 18 years and above) for this review.

### Procedure

Citations were extracted from the electronic databases and imported into the Covidence online platform (https://www.covidence.org/). After duplicates had been removed, two independent reviewers completed two rounds of screening. First, titles and abstracts were screened against the eligibility criteria and articles not meeting the criteria were excluded. For the second stage of screening, the full text of the remaining articles were sourced, and reviewed against the eligibility criteria, again by two independent reviewers. Articles which did not meet the criteria were excluded. Any disagreements were resolved by a third independent reviewer. Reference lists of included studies were screened forwards and backwards to identify further eligible studies. The full text of the articles which met the selection criteria were then moved to the next stage of the review, data extraction.

### Data extraction

A customised data extraction template was prepared, and extraction was performed independently by two reviewers. The following information was extracted from the papers for inclusion in this scoping review: author(s), year, publication details, country, study focus, population targeted, sample size and composition, QOL instrument included, and any use of accessible communication methods used in the instrument either for the items themselves, or the possible responses. Furthermore, the methods used in the development or adaptation of the instrument were extracted, including, for example, expressed use framework or disciplinary approaches, literature review or expert opinion, working groups incorporating consumers or their advocates, use of picture banks or artists, and focus groups or cognitive interviews. The extent and quality of the psychometric and validity testing of the instrument identified was extracted and categorised using the approach applied by Khadka et al. [35] and Pesudovs et al. [36] adapted from the Consensus-based Standards for the Selection of Health Status Measurement Instruments (COSMIN) (see Supplementary Information Table 2) [37]. A narrative synthesis was performed in line with the scoping review aims.

## Results

### Search results

**Fig. 1:**
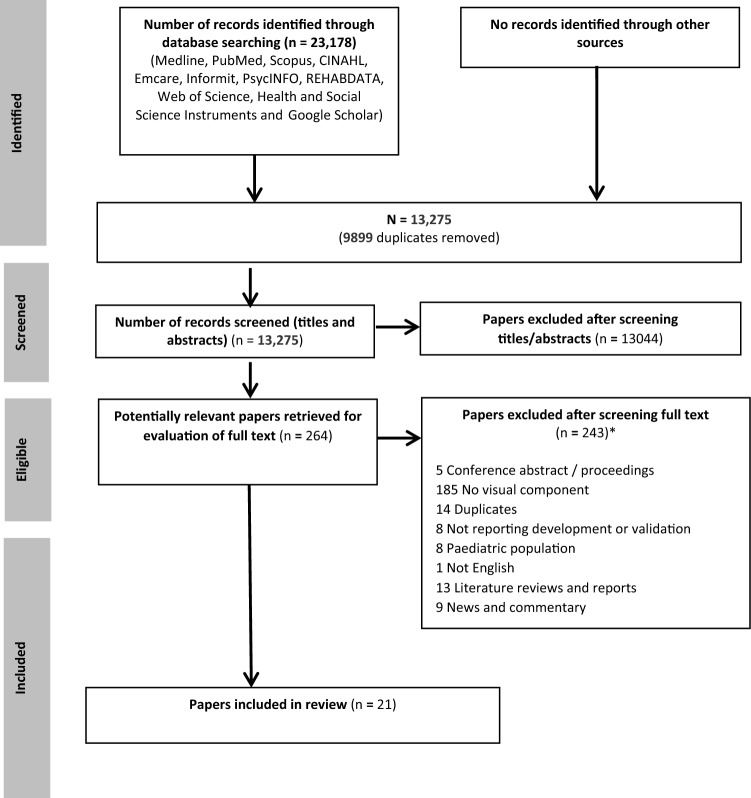
PRISMA diagram

A PRISMA flow diagram (Fig. 1) presents the systematic search results, including the identification, screening and exclusion and inclusion of identified studies. A total of 23,178 studies were retrieved from the search of electronic databases. Following removal of duplicates, 13,275 titles and abstracts were screened, with 13,044 excluded. 267 full text papers were then screened, with 246 of these excluded. The most common exclusion reasons were the instrument having no visual components or not reporting the development or validation of a QOL instrument (for example where the instrument was applied in a clinical trial) (*n* = 185). A total of 21 studies were included in the scoping review (see Additional file for a detailed summary of included studies) [38–61].

### Characteristics of included studies

Table 1 provides the overall summary of the 21 included studies. The majority were conducted in Europe (six studies), the United Kingdom (UK) (five studies), or Canada (five studies). A smaller number were conducted in the United States (US), Asia, Oceania and Africa. The earliest published study was from 2001, with the majority published from 2010 onwards. Twelve studies reported on the development of the instrument only, while seven reported a combination of development of the instrument and tests of its psychometric properties and validity. Two studies focused on the validation of the instrument only.

**Table 1:** Characteristics of included studies

Author, Year and Name of Country	QOL tool developed (if any)	Focus for study	Focus Population	Sample characteristics Size, Mean age, % women	*Based on previously published instruments*	*If yes, which previously published instruments was it based on?*
Long et al. (2008), UK	COAST	Instrument development and validation	People with aphasia	*N* = 102 Mean age = 24.5 Women (%) = 39	Yes	Stroke and Aphasia Quality of Life Scale (SAQOL-39)
Simmons-Mackie et al. (2014), Canada	ALA	Instrument development	People with aphasia	*N* = 101 Mean age = 64.59 Women (%) = 43.5	No	–
Guo et.al (2017), Singapore	ALA-C	Instrument development and validation	People with aphasia	*N* = 66 Mean age = 65.1 Women (%) = 18.2	Yes	Assessment of living with aphasia (ALA)
Engell, et al. (2003), Germany	ALQI	Instrument development	People with aphasia	*N* = 281 Mean age = 54 Women (%) = 9	Yes	Aachen Life Quality inventory (ALQI), Sickness Impact Profile (SIP)
Hilari and Byng (2001), UK	SS-QOL	Instrument development	People with aphasia	*N* = 35 Other characteristics of sample unclear	Yes	Stroke Specific Quality of Life Scale (SS-QOL)
Hilari et al., (2003), UK	SAQOL-39	Instrument validation	People with aphasia	*N* = 95 Mean age = 61.7 Women (%) = 37.3	Yes	Stroke Specific Quality of Life Scale (SS-QOL)
Whitehurst et al. (2018), UK and Canada	Name of instrument not specified	Instrument development	People with aphasia	Characteristics of sample unclear	Yes	EQ-5D-3L and EQ-5D-5L
Clark et al. (2017), USA	HRQOL-IDD	Instrument development	People with Intellectual disability	*N* = 129 Mean age = 37.9 Women (%) = 51.46	Yes	Nottingham Health Profile, Quality of Wellbeing Self Administered v. 1.04, CDC HRQOL-14, WHOQOL-Bref, Comprehensive Quality of Life Scale, Personal Wellbeing Index, Personal Outcome Scale
Turnpenny et al. (2018), UK	ASCOT-ER	Instrument development	People with Intellectual disability	*N* = 54 Age in range = 20 to over 60 Women (%) = 22	Yes	ASCOT-SCT4
Rand et al. (2020), UK	ASCOT-ER	Instrument validation	People with Intellectual disability	*N* = 264 Mean age = 93.2% are under 65 Women (%) = 46	Yes	ASCOT-SCT4
Fellinger et al. (2021), Austria	EUROHIS-QOL ESL	Instrument development and validation	People with impaired hearing and intellectual disability	*N* = 61 Mean age = 45.67 Women (%) = 37.7	Yes	EUROHIS-QOL
Buitenweg et al. (2018), The Netherlands	Name of instrument not specified	Instrument development	People with mental health conditions	*N* = 82 Mean age = 39.8 Women (%) = 48	No	–
Phattharayuttawat et al. (2005), Thailand	PTQL	Instrument development and validation	People with mental health conditions	*N* = 672 Age in range = 12–78 years Women (%) = 54.4	No	_
Erdsiek, F. (2020), Germany	PictoQOL	Instrument development	CALD population	*N* = 18, Other characteristics of sample unclear	Yes	SF-8 and WHOQOL-BREF
Hahn et al. (2004), USA	The Talking Touchscreen	Instrument development	CALD population	*N* = 126 Age in range = 21–78 Women (%) = 50.9	Yes	FACT-G (Functional Assessment of Chronic Illness Therapy General), SF-36, Standard Gamble Utility Questionnaire (SGUQ)
Hardt J. (2015), Germany	Pictorial Stark QoL	Instrument development and validation	General population	*N* = 500 Mean age = 53 Women (%) = 50	Yes	The Stark QOL
Brzoska et al. (2022), Germany	Pictorial HRQOL	Instrument development	General population and CALD	*N* = 69 Age in range = 19—61 Women (%) = 50.7	Yes	SF-36
Day, S. (2004), UK	Pictorial MYMOP	Instrument development	General population	No sample recruited	Yes	Measure Yourself Medical Outcome Profile (MYMOP)
Stothers and Macnab (2019), Canada	VSF-36 LoA	Instrument development and validation	General population	*N* = 101 Age in range = 18–93 years Women (%) = 58	Yes	SF-36
Phillipson et al. (2019), Australia	ASCOT-ER	Instrument development	Older people with cognitive impairment	*N* = 26 Mean age = 82.51 Women (%) = 57.7	Yes	ASCOT-ER
Holtmann et al. (2009), Austria, Germany and South Africa	GER-Dyzer	Instrument development and validation	Patients with Gastro-oesophageal reflux disease	*N* = 395 Mean age = 47.7 Women (%) = 48	No	_

*COAST *Communication Outcome After Stroke, *ALA *Assessment for living with Aphasia, *ALA-C *Assessment for living with aphasia—Chinese version, *ALQI* Aachen Life Quality inventory, *ASCOT-ER* Easy-read Adult Social Care Outcomes Toolkit, *EUROHIS-QOL ESL* Easy to-Understand Sign Language Version of European Health Interview Surveys, *GER-Dyzer* Gastro-oesophageal Reflux Disease Analyzer, *HRQOL-IDD* Health-Related QOL measure for people with Intellectual Disability, *Pictorial MYMOP* Pictorial Measure Yourself Medical Outcome Profile, *Pictorial HRQOL* Pictorial Health-Related Quality of Life Questionnaire, *PictoQOL* Pictorial Quality of Life, *Pictorial Stark QoL *Pictorial Stark Quality of Life, *PTQL *Pictorial Thai Quality of Life, *SAQOL-39 *Stroke and Aphasia Quality of Life Scale, *SS-QOL *Communication Accessible Version of Stroke Specific Quality of Life Scale, *VSF-36 LoA *Visual Version of the SF-36 Limitations of Physical Activities domain

Overall, nineteen different QOL instruments were identified in the studies. Thirteen were adapted from a range of previously developed instruments. This included instruments representing adaptations from existing widely applied generic QOL instruments, such as the Adult Social Care Outcomes tool (ASCOT-SCT4) [62], WHOQOL-BREF [63], 36-Item Short Form Survey (SF-36) [64], EQ-5D 3-level and 5-level versions [65, 66], Personal Wellbeing Index [67], and Nottingham Health Profile [68]. Some instruments were adapted from stroke specific QOL instruments, such as the Stroke and Aphasia Quality of Life Scale-39 (SAQOL-39) [69], Stroke Specific Quality of Life Scale (SS-QOL) [49] and Assessment for Living with Aphasia (ALA) [70]. Other instruments represented adaptations from less well known/widely applied QOL instruments (for example the Personal Outcome Scale [71], Aachen Life Quality Inventory [72], and the Measure Yourself Medical Outcome Profile (MYMOP) [65] among others. Four studies reported on communication accessible QOL instruments that were designed from first principles.

As described in Table 1, the largest proportion of the identified studies focused on people with aphasia specifically (seven studies) [38, 43, 46, 49, 53, 58, 61] and people living with a disability more broadly (generally people living with intellectual disability)(four studies) [41, 45, 57, 60]. Another three studies were focused on the general population [42, 59, 73], and two studies were focused on culturally and linguistically diverse (CALD) populations [44, 47]. One study focused on both CALD population and general population [39]. Two other studies focused on people with severe mental health conditions [40, 55], one study focused on older people with cognitive impairment [56], and one study focused on people living with gastro-oesophageal reflux disease [51]. Sample sizes ranged from *N* = 38, generally for qualitative studies or development processes, to *N* = 672 for larger scale validation studies. Two studies did not focus on reporting the results of an individual development or validation study, but rather focused on presenting a modified instrument or an overview of the methods used in its development [41, 46].

### Methods of development

The methods used in the development of the studies are summarised in Table 2. Thirteen studies (61.9%) included consumers or patients as part of the process of development of the instruments beyond inclusion as survey participants, including: six studies (28.6%)which included consumers as part of focus groups or cognitive interviews to determine the suitability of modifications to items or to develop pictures [39, 44, 52, 53, 56, 59], seven studies (33.3%) which included consumers as part of working groups; and two studies (9.5%) which included consumers as part of focus groups or cognitive interviews as well as in working groups [40, 41, 49, 57, 58, 60, 61]. Four studies (19.05%) indicated that they had included consumers in piloting the questionnaire prior to large scale validation studies [39, 46, 55, 58].

**Table 2:** Methods of development used

Author, Year, Name of Instrument	Frameworks or disciplinary approaches	Literature review/expert opinion	Working groups	Picture bank	Original drawings	Use of computer software	Iterative process	Focus groups/interviews
*Instruments for People with Aphasia*								
Long et al. (2008) COAST	–	✔	✔	–	–	–	–	–
Simmons-Mackie et al. (2014) ALA	✔	✔	✔	–	–	–	✔	✔
Guo et.al (2017) ALA-C	–	✔	–	–	–	–	–	✔
Engell, et al. (2003), ALQI	–	–	–	–	✔	–	–	–
Hilari and Byng (2001) SS-QOL	–	✔	–	–	–	–	–	✔
Hilari et al., (2003), SAQOL-39	–	✔	–	–	–	–	–	–
Whitehurst et al. (2018)	✔	✔	–	✔	✔	–	✔	✔
*Instruments for People with a Disability*								
Clark et al. (2017), HRQOL-IDD	✔	✔	–	–	–	–	✔	✔
Turnpenny et al. (2018), ASCOT-ER	–	–	✔	–	✔	–	✔	✔
Rand et al. (2020), ASCOT-ER	–	–	✔	–	✔	–	✔	✔
Fellinger et al. (2021), EUROHIS-QOL ESL	–	✔	–	–	–	–	✔	✔
*Instruments for people with mental health conditions*								
Buitenweg et al. (2018)	–	–	✔	–	–	–	✔	✔
Phattharayuttawat et al. (2005), PTQL	–	✔	–	–	✔	–	–	-
*Instruments for CALD people*								
Erdsiek, F. (2020), PictoQOL	–	–	–	–	–	–	✔	✔
Hahn et al. (2004), The talking touchscreen	–	–	–	–	–	✔	–	–
*Instruments for other populations*								
Hardt J. (2015), Pictorial Stark QoL		–	–	–	✔	–	–	–
Brzoska et al. (2022). Pictorial HRQOL Questionnaire	–	–	–	✔	–	–	✔	✔
Day, S. (2004), Pictorial MYMOP	–	–	–	–	–	–	–	–
Phillipson et al. (2019), ASCOT-ER	–	–	–	–	–	–	✔	✔
Stothers and Macnab (2019), VSF-36 LoA	–	–	–	–	✔	✔	✔	–
Holtmann et al. (2009), GER-Dyzer	–	–	✔	–	–	–	–	–

*COAST* Communication Outcome After Stroke, *ALA* Assessment for living with Aphasia, *ALA-C* Assessment for living with aphasia—Chinese version, *ALQI* Aachen Life Quality inventory, *ASCOT-ER* Easy-read Adult Social Care Outcomes Toolkit, *EUROHIS-QOL ESL* Easy to-Understand Sign Language Version of European Health Interview Surveys, *GER-Dyzer* Gastro-oesophageal Reflux Disease Analyzer, *HRQOL-IDD* Health-Related QOL measure for people with Intellectual Disability, *Pictorial MYMOP* Pictorial Measure Yourself Medical Outcome Profile, *Pictorial HRQOL* Pictorial Health-Related Quality of Life Questionnaire, *PictoQOL* Pictorial Quality of Life, *Pictorial Stark QoL* Pictorial Stark Quality of Life, *PTQL* Pictorial Thai Quality of Life, *SAQOL-39* Stroke and Aphasia Quality of Life Scale, *SS-QOL* Communication Accessible Version of Stroke Specific Quality of Life Scale, *VSF-36 LoA* Visual Version of the SF-36 Limitations of Physical Activities domain

Several approaches were used for the development of the modified instruments. Three studies (14.29%) described using theoretical frameworks for their modification of the instrument [41, 58, 61], usually drawn from the speech-language therapy literature, such as ‘Supported Conversation for Adults with Aphasia’, or ‘Aphasia-Friendly conventions’, or a ‘Total Communication Approach’ [74–76]. Eleven studies (52.38%) used an iterative process (i.e. where the instrument was developed in multiple stages with revisions made in response to feedback, which was then provided for more feedback, with more revisions made and so on. For example, initial pictorial representations were created, and these were then modified via successive rounds of feedback from consumers, family member carers and/or clinicians to further enhance and refine the pictures [39–41, 44, 45, 56–61].Twelve studies (57.13%) used focus groups or cognitive interviews to determine clarity, intelligibility, and appropriateness of modifications to instrument’s items commonly using structured approaches such as ‘think-aloud’ or ‘verbal probing’ methods [39–41, 44–46, 49, 56–58, 60, 61]. Use of literature review and expert opinion was widely used for the instruments developed for people with aphasia specifically [46, 49, 50, 53, 58, 61], and people living with another disability (such as an intellectual disability) [41, 45]. Two studies (9.52%) used existing banks of images used for communication aids (for example with people living with aphasia) as a source of suitable pictures [39, 61]. Seven studies (33.33%) developed their own images for use with an artist of other professional [43, 48, 55, 57, 59–61].

### Modification of instrument

This review identified a range of communication accessible modifications made to QOL instruments, including removal, or alteration of instrument items, use of pictures, images or picograms, editing of language following easy-read principles, modification of layout or presentation of items, or multimodal systems (incorporating audio or video elements in addition to text) to maximise consumers’ understanding of the items. All instruments included in this review modified the question items (Table 3), and all but one [53] modified the presentation of the possible response categories (Table 4). In terms of modifications to the items, the use of pictures to communicate content was widespread, with fourteen studies using line drawings or cartoons to represent or replace text describing the QOL domain [39–41, 43, 45, 47, 48, 51, 55–57].Use of black and white line drawings was common, perhaps as these allow easy and inexpensive high quality replication of the instrument via printing or photocopy. Modifications to the text of the questionnaires included: using short verbal phrases or simplifying wording; introducing additional explanations or lead-in questions; using bold text to highlight key words; or changing the formatting of the question items for example changing text size, increasing size of blank space, or presenting one question per page [39–41, 44, 46–49, 53, 55–61].

**Table 3:** Modifications to the items of the instrument

Author, year, name of instrument	Items modified (Yes or No)	Pictures use				Question layout changed	Text size changed	Wording changed	Others (describe in dot points)
		Line drawings	Cartoons	Coloured pictures	Black and white pictures				
Long et al. (2008), COAST	Yes	–	–	–	–	–	✔	✔	Item reduction
Simmons-Mackie et al. (2014), ALA	Yes	–	–	–	–	✔	✔	✔	Item reduction
Guo et.al (2017), ALA-C	Yes	–	–	–	–	–	–	✔	–
Engell, et al. (2003), ALQI	Yes	✔	–	–	–	–	–	–	–
Hilari and Byng (2001), SS-QOL	Yes	–	–	–	–	✔	✔	✔	–
Hilari et al., (2003), SAQOL-39	Yes	–	–	–	–	–	–	–	Item reduction
Whitehurst et al. (2018)	Yes	✔	–	–	–	–	✔	✔	–
Clark et al. (2017), HRQOL-IDD	Yes	–	–	–	✔	–	_	✔	Item reduction
Turnpenny et al. (2018), ASCOT-ER	Yes	–	–	–	✔	✔	✔	✔	–
Rand et al. (2020), ASCOT-ER	Yes	–	–	–	✔	✔	✔	✔	–
Fellinger et al. (2020), EUROHIS-QOL ESL	Yes	–	–	✔	–	–	–	–	–
Buitenweg et al. (2018)	Yes	✔	✔	✔	✔	✔	–	✔	–
Phattharayuttawat et al. (2005), PTQL	Yes	–	✔	–	✔	✔	✔	✔	–
Erdsiek, F. (2020), PictoQOL	Yes	–	✔	–	✔	–	–	✔	–
Hahn et al. (2004). The talking touchscreen	Yes	–	–	✔	–	✔	–	✔	–
Hardt J. (2015). Pictorial Stark QoL	Yes	–	–	✔	–	–	✔	–	–
Brzoska et al. (2022). Pictorial HRQOL Questionnaire	Yes		✔		✔	✔	–	✔	–
Day, S. (2004), pictorial version MYMOP	Yes	–	–	–	–	–	–	–	Questions added
Phillipson et al. (2019), ASCOT-ER	Yes	–	–	–	✔	✔	✔	✔	–
Stothers and Macnab (2019), VSF-36 LoA	Yes	–	–	–	–	–	–	✔	–
Holtmann et al. (2009), GER-Dyzer	Yes	✔				–	–	–	–

*COAST* Communication Outcome After Stroke, *ALA* Assessment for living with Aphasia, *ALA-C* Assessment for living with aphasia—Chinese version, *ALQI* Aachen Life Quality inventory, *ASCOT-ER* Easy-read Adult Social Care Outcomes Toolkit, *EUROHIS-QOL ESL* Easy to-Understand Sign Language Version of European Health Interview Surveys, *GER-Dyzer* Gastro-oesophageal Reflux Disease Analyzer, *HRQOL-IDD* Health-Related QOL measure for people with Intellectual Disability, *Pictorial MYMOP* Pictorial Measure Yourself Medical Outcome Profile, *Pictorial HRQOL* Pictorial Health-Related Quality of Life Questionnaire, *PictoQOL* Pictorial Quality of Life, *Pictorial Stark QoL* Pictorial Stark Quality of Life, *PTQL* Pictorial Thai Quality of Life, *SAQOL-39* Stroke and Aphasia Quality of Life Scale, *SS-QOL* Communication Accessible Version of Stroke Specific Quality of Life Scale, *VSF-36 LoA* Visual Version of the SF-36 Limitations of Physical Activities domain

**Table 4:** Modifications to the response categories of the instrument

Author, Year, Name of Instrument	Response categories modified	Pictograms	Symbols	Pictures for each levels	Wording changed	Colours/shading	Others (provide brief dot points)
Long et al. (2008), COAST	✖	✔	✔	✖	✔	✔	✖
Simmons-Mackie et al. (2014), ALA	✔	✔	✖	✔	✔	✖	✖
Guo et.al (2017), ALA-C	✔	✖	✖	✖	✔	✖	✖
Engell, et al. (2003), ALQI	✔	✔	✔	✔	✖	✖	✖
Hilari and Byng (2001), SS-QOL	✔	✖	✖	✖	✔	✔	✖
Hilari et al., (2003), SAQOL-39	✔	✖	✖	✖	✖	✖	Response scale changed
Whitehurst et al. (2018)	✔	✔	✖	✔	✔	✔	✖
Clark et al. (2017), HRQOL-IDD	✔	✔	✖	✔	✖	✖	✖
Turnpenny et al. (2018), ASCOT-ER	✔	✔	✖	✔	✔	✖	✖
Rand et al. (2020), ASCOT-ER	✔	✔	✖	✔	✔	✖	✖
Fellinger et al. (2020), EUROHIS-QOL ESL	✔	✔	✖	✖	✖	✖	Changed to interview administration
Buitenweg et al. (2018)	✔	✔	✔	✔	✖	✖	Repetitive items removed
Phattharayuttawat et al. (2005), PTQL	✔	✔	✖	✔	✔	✖	✖
Holtmann et al. (2009), GER-Dyzer	✔	✔	✔	✖	✖	✖	✖
Hahn et al. (2004). The talking touchscreen	✔	✔	✖	✔	✔	✔	✖
Erdsiek, F. (2020), PictoQOL	✔	✔	✔	✔	✖	✖	✖
Hardit J. (2015), Pictorial Stark QoL	✔	✔	✔	✔	✔	✖	✖
Brzoska et al. (2022), Pictorial HRQOL Questionnaire	✔	✔	✔	✔	✔	✖	✖
Day, S. (2004), pictorial version MYMOP	✔	✔	✔	✔	✖	✖	✖
Phillipson et al. (2019), ASCOT-ER	✔	✔	✔	✔	✖	✖	✖
Stothers and Macnab (2019), VSF-36 LoA	✔	✔	✔	✔	✔	✖	✖

*COAST* Communication Outcome After Stroke, *ALA* Assessment for living with Aphasia, *ALA-C* Assessment for living with aphasia—Chinese version, *ALQI* Aachen Life Quality inventory, *ASCOT-ER* Easy-read Adult Social Care Outcomes Toolkit, *EUROHIS-QOL ESL* Easy to-Understand Sign Language Version of European Health Interview Surveys, *GER-Dyzer* Gastro-oesophageal Reflux Disease Analyzer, *HRQOL-IDD* Health-Related QOL measure for people with Intellectual Disability, *Pictorial MYMOP* Pictorial Measure Yourself Medical Outcome Profile, *Pictorial HRQOL* Pictorial Health-Related Quality of Life Questionnaire, *PictoQOL* Pictorial Quality of Life, *Pictorial Stark QoL* Pictorial Stark Quality of Life, *PTQL* Pictorial Thai Quality of Life, *SAQOL-39* Stroke and Aphasia Quality of Life Scale, *SS-QOL* Communication Accessible Version of Stroke Specific Quality of Life Scale, *VSF-36 LoA* Visual Version of the SF-36 Limitations of Physical Activities domain

Use of pictograms to replace the potential response categories of the instrument was widespread, with all but three studies [46, 49, 50] using pictograms either alongside text or to replace text for the response categories. Use of Smiley faces (for example a frowning face for a poor level of QOL, a happy face for a good level of QOL) was common [39, 42, 43, 53, 59], but alternatives were investigated such as use of hands showing ‘thumbs up’ or ‘thumbs down’ [43], arrows or circles of increasing size [39], or cups of water/buckets of varying from empty to full of liquid [41]. An alternative approach was to use more detailed pictures relating to the expression of response levels, for example a picture of someone bed bound compared to someone walking with a walking frame, and someone walking independently to indicate different levels of mobility [39, 48, 56].

### Instrument validation

The quality and extent of validation undertaken for the identified instruments varied significantly (see Table 5). Generally, the methods for the development of the instrument were of high quality, with the intended population included, and widespread use of qualitative research or literature review and expert opinion to select items relevant to the target population. Validity and reliability testing for the instruments was notably less extensive. Thirteen of the studies did not include testing of convergent validity [39, 41, 42, 44, 45, 47, 49, 51, 56, 59–61], four included some testing of discriminant validity [48, 49, 55, 58] and only one included assessment of predictive or known group validity [46]. Seven studies included an assessment of reliability such as test-re-test agreement, interobserver or mode agreement, or [45, 46, 50, 51, 53, 58, 59].

**Table 5:** Validation of the Visual QOL tools included

Authors, year, and name of country	QOL tool developed	Sample size (*N*)	Content development				Validity					Reliability		
			Intended population	Item identification	Item selection	Unidimensionality	Convergent validity	Discriminant validity	Predictive validity	Known group	Other evidence for construct validity	Test–retest agreement	Interobserver agreement/ intermode agreement	Responsiveness
Long et al. (2008), UK	COAST	102	A	A	A	B	A	NR	NR	NR	NR	B	NR	NR
Simmons-Mackie et al. (2014), Canada	ALA	101	A	A	A	B	B	B	NR	NR	NR	B	NR	NR
Guo et.al (2017), Singapore	ALA-C	117	B	NR	B	B	A	NR	NR	B	NR	A	A	NR
Engell, et al. (2003), Germany	ALQI	281	B	NR	B	B	B	NR	NR	NR	NR	NR	NR	NR
Hilari and Byng (2001), UK	SS-QOL	35	B	B	B	NR	NR	NR	NR	NR	NR	NR	NR	NR
Hilari et al. (2003), UK	SAQOL-39	95	B	B	B	B	A	A	NR	NR	B	A	NR	NR
Whitehurst et al. (2018), UK and Canada	Name of instrument not specified	NR	NR	NR	NR	NR	NR	NR	NR	NR	NR	NR	NR	NR
Clark et al. (2017), USA	HRQOL-IDD	129	B	B	B	B	NR	NR	NR	NR	NR	NR	NR	NR
Turnpenny et al. (2018), UK	ASCOT-ER	54	A	A	A	NR	NR	NR	NR	NR	NR	NR	NR	NR
Rand et al. (2020), UK	ASCOT-ER	264	A	A	A	B	B	NR	NR	NR	B	NR	NR	NR
Fellinger et al. (2021), Austria	EUROHIS-QOL ESL	61	A	B	B	NR	NR	NR	NR	NR	NR	B	NR	NR
Buitenweg et al. (2018), The Netherlands	Name of instrument not specified	82	A	A	A	NR	NR	NR	NR	NR	NR	NR	NR	NR
Phattharayuttawat et al. (2005), Thailand	PTQL	672	A	A	A	A	A	A	NR	NR	NR	NR	NR	NR
Erdsiek, F. (2020), Germany	PictoQOL	18	B	B	B	NR	NR	NR	NR	NR	NR	NR	NR	NR
Hahn et al. (2004), USA	The talking touchscreen	126	A	B	B	NR	NR	NR	NR	NR	NR	NR	NR	NR
Hardt J. (2015), Germany	Pictorial Stark QoL	500	B	B	B	B	B	B	NR	NR	NR	NR	NR	NR
Brzoska et al. (2022), Germany	PictoQOL HRQOL	69	A	A	NR	NR	NR	NR	NR	NR	NR	NR	NR	NR
Day, S. (2004), UK	Pictorial MYMOP	NR	C	C	C	NR	NR	NR	NR	NR	NR	NR	NR	NR
Stothers and Macnab (2019), Canada	VSF-36 LoA	101	B	B	B	NR	NR	NR	NR	NR	NR	NR	A	NR
Phillipson et al. (2019), Australia	ASCOT-ER	26	B	A	A	NR	NR	NR	NR	NR	NR	NR	NR	NR
Holtmann et al. (2009), Austria, Germany and South Africa	GER-Dyzer	395	A	B	B	B	NR	NR	NR	NR	B	A	NR	A

*A* positive rating, *B* minimal acceptable rating, *C* fail/negative rating, *NR* not reported

*COAST* Communication Outcome After Stroke, *ALA* Assessment for living with Aphasia, *ALA-C* Assessment for living with aphasia—Chinese version, *ALQI* Aachen Life Quality inventory, *ASCOT-ER* Easy-read Adult Social Care Outcomes Toolkit, *EUROHIS-QOL ESL* Easy to-Understand Sign Language Version of European Health Interview Surveys, *GER-Dyzer* Gastro-oesophageal Reflux Disease Analyzer, *HRQOL-IDD* Health-Related QOL measure for people with Intellectual Disability, *Pictorial MYMOP* Pictorial Measure Yourself Medical Outcome Profile, *Pictorial HRQO*L Pictorial Health-Related Quality of Life Questionnaire, *PictoQOL* Pictorial Quality of Life, *Pictorial Stark QoL* Pictorial Stark Quality of Life, *PTQL* Pictorial Thai Quality of Life, *SAQOL-39* Stroke and Aphasia Quality of Life Scale, *SS-QOL* Communication Accessible Version of Stroke Specific Quality of Life Scale, *VSF-36 LoA* Visual Version of the SF-36 Limitations of Physical Activities domain

## Discussion

This review identified 21 QOL instruments which included an accessible communication component. Although we included QOL instruments developed for any population aged over 18 years, this review identified that the majority had been developed for use with people living with post-stroke aphasia or people living with a disability more generally (usually people living with an intellectual disability). The use of accessible communication methods in QOL instruments has been an area of focus more recently, with the majority of studies published from 2017 onwards. This relatively recent focus corresponds with the increasing emphasis in policy and practice for inclusion of consumer voices in assessing care quality and in health research more generally [15, 77–79].

As researchers have sought ways to gain the authentic involvement of people receiving health and social care services including in the evaluation of the quality of care, the diverse communication needs within populations accessing health and social care has become more apparent [80, 81]. Subsequently, the deficiencies of the ‘one-size fits all’ approach of text-based multiple-choice questionnaires, which forms most of the available PROMs, have become clearer. Researchers and practitioners in the sector have, therefore, begun to seek and develop solutions. However, the research in this area remains in its infancy. Approaches so far have generally focused on developing and validating an instrument for use with a targeted population group, for example, people living with post-stroke aphasia specifically or people living with a disability more generally.

In practice, for the application of QOL as a key quality indicator in large scale system-wide quality assessment and evaluation, communication accessible versions of QOL instruments will need to be applied consistently across diverse populations. As an example, the population using long-term care services may include older people with a range of cognitive abilities, a range of communication difficulties, and people from diverse CALD backgrounds. There is a need for rigorous development and testing processes (including qualitative and quantitative approaches) to ensure the validity of communication accessible QOL instruments by the PROM research community that can be applied with confidence across multiple diverse population groups.

Positively, there are examples of promising approaches which may form the foundations for larger scale research programs to develop communication accessible QOL instruments. There is strong evidence of the successful involvement of consumers and end users, informal family carers, care professionals and stakeholders in the development of such instruments. Successful approaches have included iterative methods where steering groups have reviewed existing QOL instruments and recommended adaptations, which were then trialled and tested in the field. Other structured approaches to involving consumers in the development process include use of qualitative think-aloud, cognitive interviewing, ‘staggered reveal’ or verbal probing approaches used as part of focus groups or in-depth individual interviews. These methods have been used successfully with people with aphasia [46, 49, 58, 61], with an intellectual disability [41, 45, 57, 60] with a mental health condition [40], people with a CALD background [44] as well as older people [56] and the general population [39], providing evidence of their broad applicability across multiple populations and communication needs.

Commonly applied modifications to existing QOL instruments have included use of pictures simplification of text, formatting changes, and use of pictograms to support responses. To date, the use of technology to facilitate the completion of QOL instruments by consumers with communication challenges has been limited. Only one study by Hahn et al., 2004 [47] developed a talking computer touchscreen with cancer patients with low literacy. With the recent prolific increase in digital capability and capacity in Australia and internationally, this is an area which may hold significant promise for the future, for example through the adaption of QOL instruments for presentation on a tablet or smart phone using video, audio or animated enhancements to support understanding and completion.

To date, no communication accessible QOL instruments have undergone extensive development and validation. Of the 21 studies identified in this review focusing on 19 different instruments, few reported validity testing of new or adapted QOL instruments. Notably, only seven studies included some assessment of the reliability of the instrument in its communication accessible form such as test-re-test agreement, interobserver or intermode agreement. Detailed evaluation of the validity of these instruments is critical. Although some have been based on instruments which have been widely used and validated previously (such as the EQ-5D-5L, WHOQOL-100 and WHOQOL-BREF, or SF-36), any modifications made to the instrument would necessitate a new validation of the modified version [6, 69]. A particularly important criterion to assess is interobserver or intermode agreement for communication accessible instruments [82]. It is likely that in any large scale application in health and/or social care systems, a proportion of the population would need to access a communication accessible version in order to facilitate self-completion. It will be important to ensure there are no systematic biases in the results obtained from different versions of the same QOL instrument, to ensure QOL assessment and reporting is valid and reliable across population groups [82–84].

## Conclusions

This review has identified a number of studies which have reported on the development and/or validation of communication accessible versions of QOL instruments. Studies identified in this scoping review demonstrate that several methods have been successfully applied e.g. with people living with aphasia post-stroke and people living with an intellectual disability, which potentially could be adapted for application with more diverse populations. A cohesive and interdisciplinary approach to the development and validation of communication accessible versions of QOL instruments, is needed to support widespread application, thereby reducing reliance on proxy assessors and promoting self-assessment of QOL across multiple consumer groups and sectors.

### Supplementary Information

Below is the link to the electronic supplementary material.

Supplementary file1 (DOCX 23 kb)
