# Supplementary material for: A scoping review of the use of visual tools and adapted easy-read approaches in Quality-of-Life instruments for adults
Source: Qual Life Res. 2023 Jun 21;32(12):3291–308. doi: 10.1007/s11136-023-03450-w (PMC10624740; doi:10.1007/s11136-023-03450-w)
Supplement: Supplementary file 1 — Supplementary file1 (DOCX 23 kb) [file 11136_2023_3450_MOESM1_ESM.docx]

**Supplementary Table 1: Keywords and search strategy in MEDLINE and Scopus**

**MEDLINE**

| # | Searches |
| --- | --- |
| 1 | Quality of life/ or Questionnaires/ or Quality-Adjusted Life Years/ |
| 2 | ("Quality of life" or QoL or hql or hqol or h qol or hrqol or hr qol or wellbeing or well-being).tw. |
| 3 | (quality adjusted life or qaly* or qald* or qale* or qtime* or preference-based instrument*).tw. |
| 4 | (quality adj2 (well-being or wellbeing)).tw. |
| 5 | (hye or hyes).tw. |
| 6 | health* year* equivalent*.tw. |
| 7 | utilit*.tw. |
| 8 | or/1-7 |
| 9 | Questionnaires/ or Self report/ |
| 10 | Health status indicators/ |
| 11 | (questionnaire* or instrument* or measures or self report* or indices or index or inventory or inventories or tool* or score* or indicator* or scale or scales or rating* or assessment or survey*).tw. |
| 12 | or/9-11 |
| 13 | Pictures/ or Audiovisual Aids/ or Comprehension/ |
| 14 | (pictogram* or pictograph* or picture* or pictorial* or easy read* or easy-read* or easy english or easy-english or simplified English or simplified-english or simplified language or simplified-language or aphasia-friendly or aphasia friendly or diagrams* or communication accessible).tw. |
| 15 | or/13-14 |
| 16 | 8 and 12 and 15 |

**Scopus**

| # | Searches |
| --- | --- |
| 1 | Quality of life* or Questionnaires* or Quality-Adjusted Life Years |
| 2 | Health status indicators* |
| 3 | Quality of life *or QoL or hql or hqol or h qol or hrqol or hr qol or wellbeing* or well-being* |
| 4 | quality adjusted life or qaly* or qald* or qale* or qtime* or preference-based instrument* |
| 5 | questionnaire* or instrument* or measures or “self report*” or indices or index or inventory or inventories or tool* or score* or indicator* or scale or scales or rating* or assessment or survey* |
| 6 | pictogram* or pictograph* or picture* or pictorial* or “easy read*” or easy-read* or “easy English” or easy-English or "simplified English" or simplified-English or "simplified language" or "simplified-language*" or "aphasia-friendly*" or "aphasia friendly*" or diagrams* or "communication accessible*" or "audio-visual aids*" or "visual aids*" |

**Supplementary Table 2:** Quality assessment criteria used for the included Visual QOL instruments (Khadka et al and Pesudovs et al adapted from COSMIN) [[42](#_ENREF_42), [44](#_ENREF_44)]

| Property | Definition | Rating | Quality criteria |
| --- | --- | --- | --- |
| Development of the instrument | |  |  |
| Intended population | The extent to which the instrument has been studied in the intended population | A | Intended population studied |
|  |  | B | Partly studied (< 50 participants) |
|  |  | C | Not studied in the intended population, only generic |
| Item identification | Selection of the item relevant to the target population | A | Comprehensive consultation with target population and experts in the area (FGD or in-depth interviews) and literature review |
|  |  | B | Minimal consultation with target population and expert opinion, and literature review |
|  |  | C | No consultation with target population |
| Item selection | Selection of the items included in the final instrument | A | A pilot instrument was developed with Rasch or factor analysis and statistically justification for removed items, and items with floor to ceiling effects removed and missing data considered. (Items removed with high proportion of missing data, > 50%; Normality tested with Kolmogorov-Smirnov or Shapiro-Wilk, or Skewness and Kurtosis values outside −2.00 to +2.00; and high proportion of ceiling effects or item end response categories of > 50%) |
|  |  | B | Only some of the above techniques were used |
|  |  | C | No pilot instruments or statistical justification of items provided in the final instrument |
| Unidimensionality | The extent to which all items fit with a single underline construct | A | Internal consistency reliability value of > 0.7 and or Rasch analysis using fit statistics (0.7-1.3) or item- trait interaction or Factor analysis (First Factor loading > 0.4 for all items) |
|  |  | B | Rasch fit statistics mostly within 0.7-1.3 range but some less well-fitting items retained, or Cronbach’s α > 0.70, and < 0.90 or factor analysis on raw scores (First Factor loading > 0.4 for all items) |
|  |  | C | Rasch analysis or Factor analysis does not support Unidimensionality or Cronbach’s α < 0.7 and > 0.9 |
| Performance of the instrument | |  |  |
| Validity |  |  |  |
| Convergent validity | The extent to which the instrument correlates with the existing instrument measuring similar construct | A | Tested against appropriate measure and correlates between 0.3 and 0.9 |
|  |  | B | Tested against a debatable choice of measure and correlates between 0.3 and 0.9 |
|  |  | C | Tested and correlates <0.3 and >0.9 |
| Discriminant validity | The extent to which the instrument diverges from other instruments that it should not be similar to | A | Tested against appropriate measure and correlates < 0.3 |
|  |  | B | Tested against a debatable choice of measure and correlates < 0.3 |
|  |  | C | Tested and correlates > 0.3 |
| Predictive validity | The extent to which the instrument can predict a future event | A | Tested against appropriate measure and correlates > 0.3 or significant difference between groups |
|  |  | B | Tested against a debatable choice of measure and correlates > 0.3 or significant difference between groups |
|  |  | C | Tested and correlates < 0.3 or no significant difference between groups |
| Other evidence for construct validity | Any other hypothesis driven testing | A | Hypothesis stated tested and proven |
|  |  | B | construct validity claimed but debatable under scrutiny |
|  |  | C | construct validity claimed but does not hold up to scrutiny |
| Reliability |  |  |  |
| Test- re- Test Agreement | The extent to which the results are repeatable when taken by the same observe | A | ICC Intra class coefficient ≥ 0.8 |
|  |  | B | ICC from 0.60 to 0.79 |
|  |  | C | ICC < 0.6 |
| Interobserver agreement/ inter-mode agreement | The extent to which results are repeatable between observers/modes of Administration | A | ICC Intra class coefficient ≥ 0.8 |
|  |  | B | ICC from 0.60 to 0.79 |
|  |  | C | ICC < 0.6 |
| A = positive rating  B = minimal acceptable rating  C= fail/negative rating  NR = Not reported | | | |
